# Supplementary material for: One-year continuation of postpartum intrauterine contraceptive device: Findings from a prospective cohort study in India
Source: PLoS One. 2024 Jun 6;19(6):e0304120. doi: 10.1371/journal.pone.0304120 (PMC11156399; doi:10.1371/journal.pone.0304120)
Supplement: S1 Table — (DOCX) [file pone.0304120.s001.docx]

**Supplemental Table S1: Comparison of Socio-demographic Characteristics between Participants Successfully Followed up and Those Lost to Follow-up**

| **Characteristic** | **Status of follow up till one year** | | **P value** |
| --- | --- | --- | --- |
|  | **Successfully followed up** | **Lost to follow up** |  |
| **State** | | | |
| Odisha | 460 (79.4) | 119 (20.5) | 0.93 |
| Chhattisgarh | 267 (79.2) | 70 (20.7) |  |
| **Age of the client** | | | |
| < = 25 years | 485 (78.2) | 135 (21.7) | 0.37 |
| > 25 years | 192 (81) | 45 (19) |  |
| **Education of the client** | | | |
| Illiterate/ just literate | 148 (71.1) | 60 (28.8) | **P < 0.01** |
| Up to 5th standard | 70 (76.9) | 21 (23.1) |  |
| 6- 12th standard | 468 (82.7) | 98 (17.3) |  |
| Graduate/Post-graduate | 37 (80.4) | 09 (19.6) |  |
| **Socio economic status** | | | |
| Lower class | 133 (74.3) | 46 (25.7) | 0.15 |
| Lower middle class | 296 (81.3) | 68 (18.7) |  |
| Middle class | 165 (80.1) | 41 (19.9) |  |
| Upper/ upper middle class | 77 (84.6) | 14 (15.4) |  |
