# Supplementary material for: One-year continuation of postpartum intrauterine contraceptive device: Findings from a prospective cohort study in India
Source: PLoS One. 2024 Jun 6;19(6):e0304120. doi: 10.1371/journal.pone.0304120 (PMC11156399; doi:10.1371/journal.pone.0304120)
Supplement: S2 Table — (DOCX) [file pone.0304120.s002.docx]

**Supplemental Table S2 – Reported number of expulsions and removals by time intervals.**

| **Time Interval since insertion** | **Outcome** | |
| --- | --- | --- |
|  | **Expulsions**  **(% of total expulsions)** | **Removals**  **(% of total removals)** |
| < 6 weeks | 64 (58.1%) | 38 (17.7%) |
| > 6 weeks to <= 6 months | 32 (29.1%) | 87 (40.6%) |
| > 6 months to <= 1 year | 14 (12.8%) | 89 (41.6%) |
| **Till one year** | 110 (100%) | 214 (100%) |
