# Supplementary material for: One-year continuation of postpartum intrauterine contraceptive device: Findings from a prospective cohort study in India
Source: PLoS One. 2024 Jun 6;19(6):e0304120. doi: 10.1371/journal.pone.0304120 (PMC11156399; doi:10.1371/journal.pone.0304120)
Supplement: S1 Appendix — (DOCX) [file pone.0304120.s003.docx]

**Data Collection Procedure**

Two tele-callers each in Odisha and Chhattisgarh were trained as ‘interviewers’ by the authors for the telephonic interactions. They were provided a 2-day training on obtaining an informed verbal consent for participation at start of each phone call, administration of the questionnaire over the telephone, and on the Standard Operating Procedures (SOPs). Each interviewer was provided with a list of phone numbers of the participants and a calendar for calling them up. The authors supervised the initial calls by each interviewer for standardization and quality assurance. Clients were followed-up by phone at around 6 weeks, 6 months and 1-year post-insertion with a date precision of about ±7 days. If we could not establish contact within one week despite more than 3 attempts at different times of the day, in the subsequent follow-up, the client was marked as ‘lost-to-follow-up’. We defined ‘Discontinuation of PPIUD’ as either removal or expulsion of the device. If a woman, after discontinuation, had not opted for any modern method of contraception till her follow-up but did not want a baby, she was deemed to have an ‘unmet need’.
