# Supplementary material for: One-year continuation of postpartum intrauterine contraceptive device: Findings from a prospective cohort study in India
Source: PLoS One. 2024 Jun 6;19(6):e0304120. doi: 10.1371/journal.pone.0304120 (PMC11156399; doi:10.1371/journal.pone.0304120)
Supplement: Attachment — Submitted filename: PLOS One comments.docx [file pone.0304120.s004.docx]

**Paper Title: One-year continuation of postpartum intrauterine contraceptive device: findings from a prospective cohort study in India**

Paper is well sequenced. Title is justifying the paper appropriately. Methods are chosen appropriately. Results and discussions are crisp and sound. The paper is recommendable.
