# Supplementary material for: One-year continuation of postpartum intrauterine contraceptive device: Findings from a prospective cohort study in India
Source: PLoS One. 2024 Jun 6;19(6):e0304120. doi: 10.1371/journal.pone.0304120 (PMC11156399; doi:10.1371/journal.pone.0304120)
Supplement: Attachment — Submitted filename: Response to Reviewers.docx [file pone.0304120.s005.docx]

**Authors’ response to reviewer comments**

We extend our sincere gratitude to the Editor and the reviewers for their valuable comments and suggestions. We have considered them with care and addressed them below for your kind perusal.

Although we have tried to include all changes suggested by the reviewers, please let us know if further modifications in the manuscript are needed.

| **Reviewer Comment** | **Author Response to Comment** | **Changes made to article** | **Page and line**  **number** |
| --- | --- | --- | --- |
| ***Reviewer 1*** | | | |
| Paper is well sequenced. Title is justifying the paper appropriately. Methods are chosen appropriately. Results and discussions are crisp and sound. The paper is recommendable. paper can be accepted as it is. | Thank you so much for your comments and appreciation. | NA | NA |
| ***Reviewer 2*** | | | |
| Thank you for your article which is interesting and shows important information regarding continuation rates of PPIUD in the two states in India. There are some parts of the paper which need the English revising. I have not done this as part of my review. | Thank you for your valuable inputs and comments. They have helped improve the manuscript a lot. We have attempted to incorporate them to the best of our capacity and hopefully the revised version meets your expectation. | NA | NA |
| Line 71-74 Correct English  In the introduction I suggest you write a paragraph on the two states involved in your study. You need to explain their social economic and geographical factors ie. are they predominantly rural or urban, are women travelling long distances to access health, what is the terrain like, are their issues like floods / mountain ranges etc… that would make access to health difficult? Are they similar states in terms of this information or do they differ. Maybe include a table to compare standard parameters? | As suggested, we have now included a paragraph about the states and their comparison to national parameters, in order to give readers a context of the study sites. | Added the below paragraph - “Odisha is a state on the eastern coast while Chhattisgarh is a state in central India. At the time of the study (2015-16), Odisha and Chhattisgarh had an estimated population of 43.1 million and 27.5 million, with 17.6% and 24.9% residing in urban areas, respectively. The total fertility rate was 2.1 and 2.2 while the modern contraceptive prevalence rates were 45.4% and 54.5%, respectively. In terms of literacy rate and poverty, in both states, the female literacy levels are below the national average (68.4%) while the proportion of population multi-dimensionally poor are much higher than the national average (24.85%). Table 1: State profiles in comparison to the national profile **Indicator** \| **Chhattisgarh** \| **Odisha** \| **India**; **Population (Census)** \| 27,571,000 \| 43,128,000 \| 12,90,235,000; **Proportion of urban population to the total estimated population (2016)** \| 24.9% \| 17.6% \| 32.8%; **Literacy rate (Female) (NFHS4)** \| 66.3% \| 67.4% \| 68.4%; **Proportion of population which is multidimensionally poor (NITI aayog (2023))** \| 29.90% \| 29.34% \| 24.85%; **TFR (NFHS 4)** \| 2.2 \| 2.1 \| 2.2; **MCPR (NFHS4)** \| 54.5% \| 45.4% \| 47.8% “ | Line 99 - 107 |
| Method There is no comment on the methodology used to insert the PPIUD. Please explain further – was it using the Kelly’s forceps, or manually, wriggles or purpose built inserter. This has an impact on expulsion rates and so needs to be explained. Also no mention of which IUDs were being inserted. Were they copper, if so which exact type. There is no mention of the counselling service provided initially to the women – was it during ANC or intra partum or post partum. Who did the counselling – designated counsellors, nurses, doctors? Was the counselling individualised or group counselling. This has a big impact on continuation rates and so needs to be explained clearly. There is no mention of the contraceptive options that women had in the post-partum period. Was it PPIUD or nothing? Or PPIUD or sterilisation? Or were other methods LARCs available like implants? Or short acting eg POP. Please explain as this has a big influence on reason for choice of method and likely reasons for discontinuation. | Thank you for this feedback. We have now included all these important details in the material and methods section. | Added the below sections –  “Under EAISI, all eligible women received family planning counselling during antenatal, intra-natal and post-partum periods by health service providers (Staff Nurses, Doctors, FP counsellors). Individualized family planning counselling was administered via one-on-one consultations. PPIUD was offered to all willing women delivering in the public health facilities after obtaining consent and due confirmation of eligibility”  “The insertion was done using the Kelly’s forceps after normal vaginal deliveries while it was done manually, before closing the endometrium in case of caesarean sections. All woman received a Copper T 380A.”  “The other post-partum family planning options available through the national family planning program during the time of the study were post-partum sterilization and condoms.” | Line 113-117  Line 121-123  Line 125-126 |
| Discussion Overall I would say the discussion portion of your paper is weak. I think if you expand more on the context and the counselling aspects of the project you may be able to discuss your results abit more meaningfully. Line 190 – English Line 188 – 192 the paragraph does not discuss anything - it’s just a repetition of the results. Please either add some discussion to this or remove.  Line 192. There is a lack of discussion regarding the expulsion rates and whether they may vary according to method of insertion. What was the method? Did the method vary between units? Please look at: [https://obgyn.onlinelibrary.wiley.com/doi/full/10.1002/ijgo.12600](https://nam11.safelinks.protection.outlook.com/?url=https%3A%2F%2Fobgyn.onlinelibrary.wiley.com%2Fdoi%2Ffull%2F10.1002%2Fijgo.12600&data=05%7C01%7CGulnoza.Usmanova%40jhpiego.org%7C33eba9001b114519f44608db3f0c601f%7C26ef7fd22a7f4135a2e4de9acf168b2a%7C0%7C0%7C638173094915850490%7CUnknown%7CTWFpbGZsb3d8eyJWIjoiMC4wLjAwMDAiLCJQIjoiV2luMzIiLCJBTiI6Ik1haWwiLCJXVCI6Mn0%3D%7C3000%7C%7C%7C&sdata=cxAMy7QC64Ym0O%2F6XuhIt7I44ZykL4IJxGG8x0888P4%3D&reserved=0) One of the countries is India. Expulsion rates are different according to type of insertion. If you don’t have the data to look at this ten you must mention this as a limitation in the discussion. | Thank you very much for your valuable inputs on the discussion section. We have attempted to incorporate them.  Line 190: We have reframed the sentence.  Line 188-192 – In the first paragraph of the discussion we have summarized the key results with reference to our study objectives. This is in line with the STROBE checklist/guidelines for reporting of observational studies.  As has now been detailed out, mostly all insertions were done using modified Kelly’s forceps, following a vaginal delivery. Very few insertions were done manually after caesarean sections. Only 15 of the 916 cases were of manual insertions after a caesarean section, and none of them reported an expulsion. Therefore, we were not able to include intra caesarean category for ‘type of insertion’ variable in the regression model for expulsion – we have now included this as part of the discussion.  In addition, as has been shown in table 5, the expulsion rates did not vary by cadre of provider or by timing of insertion. We have now added some discussion points around this finding. | Reframed first paragraph of discussion section is as below –  “We report that continuation rate of PPIUD was 88.7% at 6 weeks, 74.8% at 6 months and 60.1% at 1 year. It dropped maximally within the first 6 weeks of insertion. Among those who got the PPIUD removed, almost half reported associated side effects like bleeding, pain in abdomen and vaginal discharge, to be the primary reason. On conducting Cox proportional hazards modeling, woman’s education status and experiencing associated side-effects emerged as significant determinants of PPIUD removal; we could not find any significant determinant for PPIUD expulsion.”  Added discussion around expulsion vs method of insertion –  “ It is encouraging to note that PPIUD expulsion rates were not significantly impacted by provider profile (e.g., doctor, nurse). This finding corroborates with previous studies that have found no significant difference between expulsion rates in insertions done by doctors and nurses, or significantly fewer expulsions when insertions were done by nurses versus the doctors [22, 28]. This gives confidence in FP task shifting and sharing to providers and facilities further downstream, which can further improve access to PPIUD services. A significant proportion of institutional deliveries in India are conducted by nurses and midwives (including auxiliary nurse midwives and general nurse midwives) [10, 29] and therefore they can play a key role in enabling access to PPIUD services, in light of these findings.  The study also found no significant differences in expulsion rates by type of insertion, a finding which is corroborated by earlier studies [22,30]. It is worth noting that we could not include manual placement following caesarean section as a category for the ‘type of insertion’ variable, as there were very few (15/916) such cases and none of those insertions had an expulsion, as reported till the last follow up. “ | Line 222- 228  Line 253-266 |
| IN paragraph starting line 194, you say the discontinuation rates are similar to what has been reported before. Perhaps this is true in India but necessarily true elsewhere. Please see this paper from Tanzania: [https://reproductive-health-journal.biomedcentral.com/articles/10.1186/s12978-020-00999-4](https://nam11.safelinks.protection.outlook.com/?url=https%3A%2F%2Freproductive-health-journal.biomedcentral.com%2Farticles%2F10.1186%2Fs12978-020-00999-4&data=05%7C01%7CGulnoza.Usmanova%40jhpiego.org%7C33eba9001b114519f44608db3f0c601f%7C26ef7fd22a7f4135a2e4de9acf168b2a%7C0%7C0%7C638173094915850490%7CUnknown%7CTWFpbGZsb3d8eyJWIjoiMC4wLjAwMDAiLCJQIjoiV2luMzIiLCJBTiI6Ik1haWwiLCJXVCI6Mn0%3D%7C3000%7C%7C%7C&sdata=hKi4B6jP2s%2B9ZOBPDFk3cnMaO9qlac%2FJ5nyiMtinu%2FA%3D&reserved=0) I would argue your discontinuation rates are quite high and it worrying that such a large proportion of those who discontinued do not take up any modern contraception. I think you need to discuss this further. Is this because there is no other reliable method available free of charge? Or is this because they wanted to have more children and so don’t want any contraception, or is this because the side effects were so awful that they have been put off using any method of contraception. You don’t mention consent for PPIUCD. Was this taken properly? Or could the IUCD have been inserted without proper consent. Although you mention complex issues I think you need to go in depth a little more. I would add a paragraph explaining the context better – is contraception widely accepted in these areas of India? Or are there cultural barriers, do husbands approve generally, are women empowered to make decisions about contraception or do their husbands/mothers-in-law make that decision. All these aspects need to be mentioned and discussed. If you don’t have the data or cant find anything in the literature then you have to put this in your discussion as a weakness and suggest further qualitative studies need to be undertaken to understand exactly why the discontinuation rates are so high. This paper looks at counselling aspects and acceptance in the first place of PPIUD: [https://obgyn.onlinelibrary.wiley.com/doi/full/10.1002/ijgo.12599](https://nam11.safelinks.protection.outlook.com/?url=https%3A%2F%2Fobgyn.onlinelibrary.wiley.com%2Fdoi%2Ffull%2F10.1002%2Fijgo.12599&data=05%7C01%7CGulnoza.Usmanova%40jhpiego.org%7C33eba9001b114519f44608db3f0c601f%7C26ef7fd22a7f4135a2e4de9acf168b2a%7C0%7C0%7C638173094915850490%7CUnknown%7CTWFpbGZsb3d8eyJWIjoiMC4wLjAwMDAiLCJQIjoiV2luMzIiLCJBTiI6Ik1haWwiLCJXVCI6Mn0%3D%7C3000%7C%7C%7C&sdata=YJ1QpSLETEQO4yCbT06knrTOxSO815bVy%2FwI9SWoDRE%3D&reserved=0) | We compared the expulsion rates reported at the time of study. Thanks for Tanzania paper, its interesting and captures interesting finding. Please note that while as per law, women are the sole decision makers as far as contraception goes in India, social factors as mentioned by you definitely play a role. We have now included this in our discussion. As part of the program, before insertion of PPIUDs, consent was taken by the provider – we have added this detail in the methods section.  Method switching was not a focus of our study and definitely need more research and we agree with your observation – we have added a suggestion on conducting qualitative studies to delve deeper into this subject.  Although the continuation rates are comparable to other studies from India, analyzing the reasons for removals does point towards a possible gap pertaining to the quality of counseling on the method. Majority of those who got the device removed reported expected side effects like vaginal discharge and pain in abdomen to be the primary reasons for opting for removal. This aligns with findings of earlier studies which too reported expected side effects to be the primary reason for removal. This emphasizes the need to include discussion on these transient and expected side effects during the counseling on the method and prepare women for it. This could help further improve continuation rates of the method. It is also important to highlight that around 5% clients reported pressure from their partners as the primary reason for discontinuing the method. Some recent studies from India have also pointed out family pressure to be an important reason for removal of PPIUCDs. This emphasizes the need to involve partners and even family members like mother in laws in the discussions around family planning during different stages of the pregnancy.  The lack of switching among those who discontinued the method (either because of expulsion or removal), could also be because of limited options available for post-partum contraception in India’s FP program – at the time of the study only two other options- either condoms or PPS – were available for post-partum FP. It may also point towards the fact that those who experienced the side effects (majority of removals were due to experiencing side effects), may not have immediately wanted to switch to another method. It should be acknowledged that the status of switching among discontinuers was limited by the fact that they were not followed up further once they had reported discontinuation of the method in any of their scheduled follow ups – hence women who would have taken some time to switch may not have been captured. We have now included all these aspects in the discussion section, citing relevant literature. | Added sections to discussion section –  “However, the study also does highlight that a majority of participants who got the device removed, reported expected side effects like discharge and pain in abdomen as the primary reason for removal. This corroborates with findings from other studies conducted in India [10, 24, 25] and emphasizes the need to include discussion on these expected side effects during the counseling. There are studies from India and other low- and middle-income countries, which have shown gaps in the quality of family planning counseling, specifically with regards to discussion on expected side effects of methods [26, 27].”  “At the time of the study, only three options- Condoms, PPIUDs or post-partum sterilization were available for post-partum FP at the selected public health facilities.  The lack of switching may also point towards the fact that those who had removals because of the side effects (majority of removals were due to experiencing side effects), may have preferred a gap before switching to another method. It must be acknowledged that the assessment of switching among discontinuers was limited by the fact that they were not followed up any further once they had reported discontinuation – hence women who would have taken some time to switch after discontinuation, may not have been captured. “  “The non-linear role of education in PPIUD continuation calls for further exploration. Education shapes health and care seeking related attitudes, intentions and behaviors.[31,32] However, female education and its manifestations in LMIC settings is an outcome of complex inter-linkages between socio-cultural milieu and other structural determinants of overall societal development and gender empowerment. PPIUD uptake and continuation among women in LMIC contexts have been shown to require multi-layered and multi-dimensional interventions.[33] Many studies from India have highlighted the key role of partners and family members in the decision to take up and use a family planning method [25, 34, 35]. Our study also found that some participants opted for removal primarily because of their partner’s/husband’s disapproval. This emphasizes the need for increased engagement with family and partners during counseling. “ | Line 231- 237  Line 240-247  Line 267-277 |
| Line 225 The loss to follow up proportion at 1 year follow up is quite high (60%). Please make loss to follow up at different stages more explicit, perhaps in a table. You also need to look at the demographics of those lost to follow up to see if they are different to the overall group so that you can estimate if there may be a degree of bias in your analysis due to this. Please include this. Line 229 – 231 What do you mean? Did you ask all those who discontinued to come to a health centre to have face to face follow up? If so, you need to add this in your methodology and talk about the results of these follow ups. If you don’t have the results of these follow up appointments then you cannot say they validate your findings. | We would like to clarify that the loss to follow up at one year was 20.6%. This has been corrected in the discussion section (where we are including this as a limitation) and has also been stated in the initial part of the results section. We would also like to add, that as has been mentioned in the sample size estimation paragraph, even if our study had seen a 50% lost to follow up rate, the sample size would have been sufficient.  As suggested, we have now included numbers on loss to follow up at each stage, in the newly added table no. 2.  Distribution of clients with respect to follow up status in the study –   1. Outcome known at one year – 727 (79.4%) 2. Lost to follow up prior to 6 months (at 2^nd^ follow-up) – 83 (9.06%) 3. Lost to follow up after 6 months (at 3^rd^ follow-up) – 106 (11.57%)   We have compared the demographics of those lost to follow up versus those who participated throughout (Supplemental table 1) and found no significant differences by location, age and SE status. However, there was a significant difference with respect to education, with higher proportion of respondents who were lost to follow up being lesser educated. This may have influenced the study estimates of continuation rates (discontinuation rates could be higher) as the study did find education status as a significant determinant of removals. We have now included this as a limitation in the discussion section.  Line 229-231 – We agree. We have clarified the context now. All those who had reported discontinuation during their follow ups were encouraged to follow up with health service providers at the health facility, post completion of data collection interview. These follow ups were not a part of the study and as suggested, we have reframed the sentence to ensure it does not reflect a data validation exercise. | Added to the discussion section below –  “All recruited acceptors could not be followed-up as some telephone numbers were invalid, some could not be reached and some did not respond during subsequent follow-ups (20.6% clients were lost-to-follow-up by the 3rd follow-up). On comparing the background characteristics of those who were lost to follow up versus those who were successfully followed up, we did find a significant difference in their education status. Higher proportion of women who were lost to follow up during this study, had lesser education when compared with women who could be successfully followed up. This may have had an influence on the estimation of the PPIUD continuation probability, as education status did emerge as a significant risk for PPIUD removals in our study.” | Line 291-299 |
| Table 1 Type of insertion – please explain your categories: post placental and postpartum. | Postplacental insertion refers to insertion performed within 10 min of expulsion of placenta  following a vaginal delivery whereas postpartum insertion is done after 10 min but within 48 h of delivery.  We have added this to the manuscript. | Added in the methodology –  “ IUD insertion which was performed within 10 minutes of expulsion of placenta was regarded as post-placental insertion while those performed after 10 minutes but within 48 hours of delivery were regarded as immediate post-partum insertion. “ | Line 160-162 |
| Table 2 How can your continuation rate be 60.4% overall if your expulsion and removal rates are 13.7% and 29.9% respectively (totalling 43.6% which would give you a continuation rate of 56.4??). The maths doesn’t add up. Please explain. | As we had used the life table analysis method, these are probabilities of each event (continuation, removal or expulsion) happening at specified time intervals along with their confidence intervals. They may therefore not add up to 100%.  In order to avoid any confusion regarding this, we have now kept only the life table analysis for continuation (newly added table 2)in the main manuscript. This also depicts lost to follow up status at different intervals. We have included details on number of removals and expulsions in the narrative and also added a supplemental table 2 pertaining to it. | Table 2  Supplemental table 2 | NA |
| Table 5 Why didn’t you look at expulsion following intra caesarean insertion. There are studies showing expulsion rates are lower following intra-caesarean insertion compared to within 10 mins of a normal vaginal delivery. Please explain why not or add analysis. | As has been previously explained, the sample includes very few PPIUCDs that were inserted after a caesarean section and therefore we are unable to comment on expulsion rates following caesarean sections. The study was conducted in sub-district level facilities and as women requiring caesarean sections are usually referred to district or higher level facilities (very few sub-district level facilities conduct caesarean deliveries), the sample consists of very few post caesarean section PPIUCDs.  We have added this to the discussion section. | Added to the discussion section –  “It is worth noting that we could not include manual placement following caesarean section as a category for the ‘type of insertion’ variable, as there were very few (15/916) such cases and none of those insertions had an expulsion, as reported till the last follow up“ | Line 263 - 266 |
