# Supplementary material for: One-year continuation of postpartum intrauterine contraceptive device: Findings from a prospective cohort study in India
Source: PLoS One. 2024 Jun 6;19(6):e0304120. doi: 10.1371/journal.pone.0304120 (PMC11156399; doi:10.1371/journal.pone.0304120)
Supplement: Attachment — Submitted filename: AuthorReplytoEditorComments.docx [file pone.0304120.s006.docx]

| **S.no.** | **Editor Comment** | **Author response to the comment** | **Line number/Section**  **(In the revised version)** |
| --- | --- | --- | --- |
| 1. | Introduction section needs expansion with background, context/need of the study and framed objectives accordingly. | We have expanded the background section, clearly bringing out the context and the need for this study. We have specified the primary and secondary objectives of the study towards the end of the background section. | Introduction section |
| 2. | Data and Method section needs improvements. The table number 1 and their interpretation should be move to result part as an starting section. | As was suggested by one of the reviewers, we had introduced this table in the methods section to describe the study settings in more detail. This is not a finding from the study but a detailed description of the state profile and its comparison to the national profile – hence we feel it is more appropriate in the methods section describing the study setting. We have corrected the table numbers in the results section as suggested. | Methods & Results section |
| 3. | Although there details about the sample size and design including sample framework used in the selection of samples but it should be very clearly mentioned under the sub-heading of 'sampling design and sample size'. | As suggested, we have now divided the methods section into different sub-sections with Study design, sampling and sample size being one of them. | Methods section |
| 4. | The paragraphs in this section are large needs to divide into smaller paragraph with lined and readable flow. | As suggested we have now divided the methods section into different sub-sections. We have also further divided the larger paragraphs into smaller paragraphs for improved readability. | Methods section |
| 5. | The data collection procedure can be moved to the appendix part of the manuscript. | As suggested, we have moved the data collection procedure to an appendix (S1 Appendix). | Appendix S1 |
| 6. | The statistical methods mentioned in line number 176-184 needs to revise and strengthen. As on the modelling part authors mentioned about using Cox modelling needs appropriate scientific exploration and valid justification for the use of the such hazard modeling procedure for this study. | As suggested, we have now strengthened the statistical analysis section detailing out the process of estimating cumulative continuation probabilities as well as adding a justification for using Cox proportional hazards modelling. The justification added is as following – ‘Cox proportional hazards models are recommended in analysis of prospective studies which have a follow up period during which occurrence of events is observed. This is because they have more statistical power than other models as they account for time until events occur.’ | Line 212-215 |
| 7. | Ethical Clarence should be in under separate heading. | As suggested, it is now under a sub section on Ethics. | Line 224- 230 |
| 8. | In the result section the description of the tables seems very loosely written needs proper interpretation. | We have done interpretation of the results in the Discussion section, which is as per various guidelines on writing different sections of a scientific manuscript. In the results section, we have narrated the key findings from each table. | NA |
| 9. | Table number also needs correction as table no 1 is in method section as well as in the result section. In the table 1 of result section, change the word client to respondents. What methods the authors have followed to classify the respondents into various socio-economic status category. | We have corrected the table numbers.  As suggested, we have changed the term to respondents.  The BG Prasad scale was used for classifying respondents into various socio-economic status categories. The same has been mentioned in the methods section (Line 187 in clean version) | Methods & Results section |
| 10. | Heading of table number 2- 'life table'- what does it mean for this specific table. The word should be changed with the appropriate words which should reflect the actual meaning. | We used Kaplan-Meier time-to-event probabilities to estimate the cumulative continuation rates. It utilizes all follow-up time contributed by the participants, including time to discontinuation (due to expulsion or removal) as well as incomplete follow-up time as censored event time. We used self-reported time of removal or expulsion, together with date of insertion, to calculate the time to event in days. We considered self-reported removals and expulsions as discontinuation events, regardless of whether the woman then took up a similar or different contraceptive method. We censored participants who had not reported discontinuation at their last completed follow up – for participants who were categorized as lost to follow up, censoring time was kept as the time interval between date of insertion and date of last follow up; for participants who reported continuing with the method at one year follow up, the censoring time was kept as 365 days.  As suggested, we have now replaced the term ‘Life table analysis’ with ‘Cumulative continuation rates.’ We have added the above details in the Statistical Analysis section. | Table 2 |
| 11. | The sample size varies in different tables which should be clearly mentioned in sample design section of under the methodology part of the manuscript. | The sample size varies for tables which further analyse expulsions and removals – like reasons for removal and switching after either expulsion or removal. For such tables, the sample consists of study participants who reported these events from among all participants. We have included this information in the narrative of results section.  We have now also added a paragraph in the statistical analysis section regarding the same. | Result section & Line 219 – 221 of Methods section |
| 12. | The discussion section also needs restructuring with clear cut findings of the study followed by the suggestive measures as the study seems programme based outcome linked to FP and unmet needs which remained an important concern before the government and programme strategic concern. That would be worth of the study and knowledge addition of the study. | As suggested, we have re-structured the discussion section to bring the suggestive measures after discussion on key findings of the study.  We have separated out the paragraph which discussed the strengths and limitations of the study, and kept it at the end. | Discussion section |
| 13. | Limitations of study is although mentioned in discussion section but needs proper paragraph. | As suggested, we have now kept a separate paragraph on ‘Strengths and Limitations’ in the latter half of the Discussion section. | Line 335 – 363 of Discussion section |
| 14. | Careful editing is needed of the whole manuscript. | We have re-read and made some additional copy-edits. Hope this meets the expectation. | NA |
